# Surface Characterization of Mesoporous CoO_x_/SBA-15 Catalyst upon 1,2-Dichloropropane Oxidation

**DOI:** 10.3390/ma11060912

**Published:** 2018-05-29

**Authors:** Elisabetta Finocchio, Jonatan Gonzalez-Prior, Jose Ignacio Gutierrez-Ortiz, Ruben Lopez-Fonseca, Guido Busca, Beatriz de Rivas

**Affiliations:** 1 Dipartimento di Ingegneria Civile, Chimica e Ambientale (DICCA), Università degli Studi di Genova, Via all’Opera Pia 15, I-16145 Genova, Italy; guido.busca@unige.it; 2 Departamento de Ingeniería Química, Facultad de Ciencia y Tecnología, Universidad del País Vasco UPV/EHU, P.O. Box 644, E-48080 Bilbao, Spain; jonatan.gonzalez@ehu.es (J.G.-P.); joseignacio.gutierrez@ehu.es (J.I.G.-O.); ruben.lopez@ehu.eus (R.L.-F.)

**Keywords:** surface characterization, FT-IR spectroscopy, cobalt oxide, SBA-15, Cl-VOC oxidation

## Abstract

The active combustion catalyst that is based on 30 wt % cobalt oxide on mesoporous SBA-15 has been tested in 1,2-dichloropropane oxidation and is characterized by means of FT-IR (Fourier transform infrared spectroscopy) and ammonia-TPD (temperature-programmed desorption). In this work, we report the spectroscopic evidence for the role of surface acidity in chloroalkane conversion. Both Lewis acidity and weakly acidic silanol groups from SBA support are involved in the adsorption and initial conversion steps. Moreover, total oxidation reaction results in the formation of new Bronsted acidic sites, which are likely associated with the generation of HCl at high temperature and its adsorption at the catalyst surface. Highly dispersed Co oxide on the mesoporous support and Co-chloride or oxychloride particles, together with the presence of several families of acidic sites originated from the conditioning effect of reaction products may explain the good activity of this catalyst in the oxidation of Chlorinated Volatile Organic Compounds.

## 1. Introduction

The catalytic oxidation of Chlorinated Volatile Organic Compounds (Cl-VOC) is a complex process, which is affected not only by the redox properties of the catalyst, but also by its structural characteristic and surface acid-basic properties. Catalysts showing high Bronsted acidity, such as zeolites and zeolite-supported metals, have been widely tested in the oxidation of chlorinated compounds, such as chlorinated alkanes and alkenes. These catalysts, while favoring the oxidation process through an improved adsorption of chlorinated compounds, are also more prone to deactivation by coke deposition or heavy by-products formation, affecting the strongest acidic sites [1,2,3,4,5,6]. Moreover, HCl formed during the reaction attacks the zeolite structure, leading to dealumination and consequent loss of acidity. The catalyst activity can be at least partially recovered by regeneration in humid air, which restores surface hydroxyl groups and gasifies coke to an appreciable extent, but deactivation by HCl tends to be irreversible [7,8,9,10]. On the other side, Lewis acidity has been also reported to favor chlorinated compounds adsorption (i.e., through Cl atom) and reaction intermediates adsorption, thus enhancing the deep oxidation of adsorbed species [11,12,13,14,15].

Catalyst porosity also plays and important role by controlling the morphology of a supported active phase and by favoring the adsorption/desorption steps of Volatile Organic Compounds, as reported for mesoporous silica-based catalysts and adsorbents [16,17,18,19]. Recently, we reported on the activity of a mesoporous Co-SBA system in 1,2-dichloroethane (DCE) combustion. A family of catalysts in the composition range 10–50 wt %Co_3_O_4_ has been tested, showing appealing results in terms of low T_50_ temperatures (330–340 °C for samples 30–50 wt % Co_3_O_4_) and high selectivity to CO_2_ and HCl (90% DCE conversion to CO_2_ in the range 365–380 °C for samples 30–50 wt % Co_3_O_4_). These catalysts are characterized by the distribution of the cobalt oxide phase partly within the SBA pores. Namely, at the highest cobalt oxide contents, large CoO_x_ particles are located on the external surface, while the smallest particles are encapsulated within the mesoporous channels, enhancing the dispersion of the active phase and therefore improving redox properties. Acidic silanol groups of the mesoporous silica support and Lewis acidic sites arising from Co introduction are exposed at the surface in all the composition range studied (10–50 wt % Co_3_O_4_) [20]. As a result, both mesoporosity and an optimal acidic sites distribution appear to be key parameters in order to, on one hand, provide good morphological properties, and, on the other hand, strongly adsorb chlorinated reactants, which become more prone to oxidative attack, and avoid deactivation phenomena, such as heavy coking.

In this work, we present an FT-IR study of surface acidity 30Co/SBA catalyst (CoO_x_ on SBA-15) in different steps of the 1,2-dichloropropane (1,2DCP) conversion process. This specific catalyst formulation has been chosen for further IR investigations, as it provides a good balance between the lowest amount of CoO_x_ phase and a noticeable oxidizing activity in chlorinated compound conversion. This study was supplemented not only by the definition of the corresponding light-off curve in a fixed bed reactor, but also by an extensive characterization of the used sample by BET (brunner−emmet−teller) measurements, ammonia adsorption, and elemental analysis.

## 2. Materials and Methods

The CoO_x_ catalyst supported on SBA-15 (30Co/SBA, 31.3 CoO_x_ wt %) was prepared by the wet impregnation of silica under reduced pressure, using an aqueous solution of cobalt (II) nitrate (98%, Aldrich, Darmstadt, Germany). The mesoporous silica support has been synthesized in acidic conditions using a mixture of Pluronic P123 triblock copolymer (EO20PO70EO20, Aldrich, Darmstadt, Germany) as template and tetraethyl orthosilicate (TEOS, 98%, Aldrich, Darmstadt, Germany), aged in a stainless-steel autoclave under static conditions for hydrothermal treatment. The resulting product, after drying, was calcined in flowing air at 550 °C for 6 h. The fresh SBA-15 support shows a surface area of 743 m^2^·g^−1^, lowered to 440 m^2^·g^−1^ after impregnation. More details on catalyst morphology are reported in reference 20 and are summarized in the following paragraphs.

In this sense, we examined the effect of Co_3_O_4_ loading (in the range 10–50 wt %) in a series of SBA-15 supported samples on the oxidation of chlorinated hydrocarbons using 1,2-dichloroethane as a model compound. The formulation having the lowest cobalt oxide content (i.e., 10 wt %) showed a poor activity, and total conversion of 1,2-dichloroethane was not reached up to 500 °C. Conversion to CO_2_ was found to increase with oxide content up to 30 wt %. For higher loadings, no marked promotion of the catalytic behavior was noticed. The catalyst was tested in 1,2-dichloropropane (1,2DCP) conversion in a fixed bed PID Eng&Tech flow reactor (PID Eng&Tech, Madrid, Spain), at the following conditions: 1000 ppm 1,2DCP in dry air, gas flow rate 500 cm^3^·min^−1^, space velocity 30,000 h^−1^, 150–500 °C. Reactants and products analysis has been carried out by a GC7980 Agilent gas chromatograph (Agilent, Santa Clara, CA, USA), which was equipped with TCD (thermal conductivity detector) and ECD (electron capture detector detectors).

FT-IR surface characterization study has been performed using pure powder disks (20 mg weight average) for adsorption studies, always working in transmittance mode. Samples have been characterized by pyridine adsorption and desorption before and after the reaction of 1,2 dichloropropane directly in the IR cell. A typical adsorption-reaction experiment is designed as follows: Activation of the pure powder disk in air and in vacuum at 500 °C, adsorption of 0.67 kPa 1,2DCP at room temperature, heating by steps up to 500 °C with or without air in the IR cell. After heating at 500 °C, or at intermediate temperatures, the surface of the catalyst has been outgassed at room temperature and pyridine adsorption and subsequent desorption has been carried out. Blank experiments that were carried out in the IR apparatus have revealed that 1,2DCP conversion was significant only at 500 °C, leading to the formation of chloropropenes and HCl. IR spectra were recorded on a Thermo Nicolet Nexus FT-IR instrument (Thermo Fisher Scientific, Waltham, MA, USA) in the range of 4000–400 cm^−1^ (DTGS detector, 100 scans).

## 3. Results and Discussion

### 3.1. Activity Results and Characterization of the Fresh and Spent Catalyst

**Figure 1:**
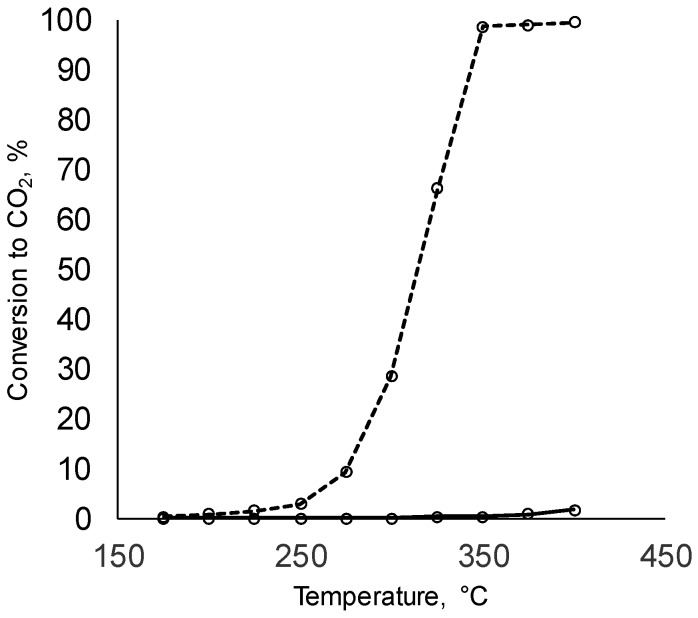
Light-off curve of CO_2_ production from 1,2DCP combustion. Dashed line: 30/CoSBA. Full line: SBA-15 support.

In order to better rationalize the obtained findings from the spectroscopic study, some results on the performance of the investigated SBA-15 supported cobalt catalyst in the oxidation of 1,2-dichloropropane will be briefly discussed. These results are focused on the characterization of the sample before and after a catalytic test using a fixed-bed flow reactor between 150–500 °C. In Figure 1, the light off curve of CO_2_ production is reported for the 1,2DCP conversion over 30Co/SBA and over the bare support. Pure SBA-15 does not show any relevant CO_2_ formation up to 450 °C. Indeed, addition of cobalt oxide is fundamental to reach total conversion to CO_2_: The oxidation activity starts to be significant above 250 °C (T_50_ = 320 °C) and is almost total above 350 °C (T_90_ = 350 °C). These temperature levels were slightly lower than those noticed for the oxidation of 1,2-dichloroethane [20]. This increased reactivity for 1,2DCP can be related to the structure of the organic substrate, having a secondary C–Cl group. We already reported on the easier activation of a secondary C–Cl group in comparison with a primary C–Cl group in dehydrochloriantion reaction, through the formation of adsorbed intermediate carbenium-like species, as discussed below. Such a species should be more prone to deep oxidation, and following the formation of oxygen-containing fragments [1,14].

The fresh sample shows a specific surface area of 440 m^2^·g^−1^, a pore volume of 0.59 cm^3^·g^−1^ and a pore diameter of 58 Å. The bimodal pore distribution of the SBA support is kept also after cobalt deposition. The corresponding isotherms and textural properties of the samples are included in Figure S1 and Table S1. Cobalt oxide nanocrystallites are highly dispersed with an average size of 14 nm, as determined by XRD (Figure S2) [20]. As discussed before, the dispersion of the active phase due to the interaction with the silica matrix improved the redox properties of the catalyst, increasing the reducibility as long as the particle size decreased. The overall acidity (135 μmol_NH3_·g^−1^, corresponding to 0.31 μmol_NH3_·m^−2^) was evaluated from the adsorption of ammonia at 100 °C, followed by thermogravimetry.

After the reaction test, the used catalyst shows a significantly lower surface area (353 m^2^·g^−1^), a slightly lower pore volume (0.53 cm^3^·g^−1^), and a roughly similar pore diameter (55 Å). On the other hand, the acidity increased very slightly up to 141 μmol_NH3_·g^−1^ (i.e., 0.4 μmol_NH3_·m^−2^). This larger overall population of acid sites should be related to the presence of chlorine at the surface, about 0.4 wt %, as determined by SEM-EDX (scanning electron microscopy with energy dispersive X-ray spectroscopy) analysis. Furthermore, this analysis also revealed that chlorine was preferentially present in the vicinity of the cobalt oxide, rather than on the surface of the silica support.

### 3.2. Surface Characterization

Surface acidity has been studied by probe molecules adsorption over the fresh catalyst and the blank support, and after several pretreatments in the presence of 1,2DCP at different temperatures.

**Figure 2:**
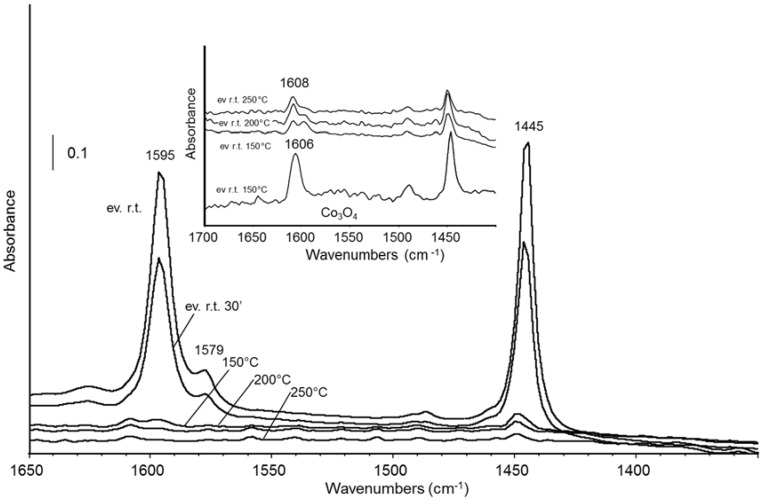
FT-IR subtraction spectra of surface species arising from pyridine adsorption over fresh 30Co/SBA catalyst. Inset: magnification of spectra recorded at high temperature. The spectrum of pyridine adsorbed over the reference Co_3_O_4_ has been reported for comparison.

The spectra of surface species arising from pyridine adsorption and desorption at an increasing temperature over the fresh 30Co/SBA sample are reported in Figure 2. The main bands due to stretching vibrations of pyridine adsorbed over silica appear at 1595, 1579, and 1445 cm^−1^, all of them being assigned to pyridine weakly interacting with silanols through H-bonds [21,22]. Correspondingly, in the high frequency region, the band related to isolated silanol groups centred at 3745 cm^−1^ is strongly perturbed. After outgassing at 150 °C, bands due to weakly adsorbed pyridine are strongly reduced in intensity. Consequently, components at 1608 and 1450 cm^−1^, became more evident. These features are due to ring stretching modes of pyridine molecules coordinated over Lewis sites of medium-weak intensity, for instance, exposed Co ions [20,23]. These features are detected up to 250 °C (Figure 2, inset). The spectrum of pyridine interacting with pure Co_3_O_4_ is reported for comparison in the same inset. As expected, pyridine adsorption on pure SBA-15 shows features typical of H-bound species, readily disappearing upon outgassing (spectra not reported).

**Figure 3:**
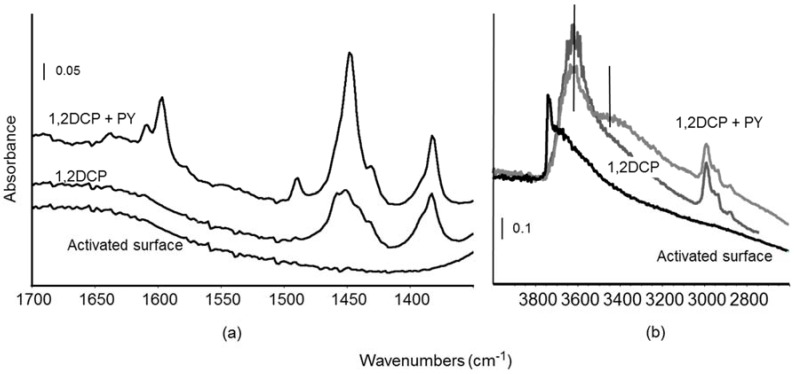
FT-IR spectra of surface species arising from 1,2DCP adsorption and heating at 50 °C and pyridine-1,2DCP coadsorption over the fresh 30Co/SBA catalyst (**a**); High frequency region (**b**).

Adsorption of 1,2DCP at room temperature and after a short heating at mild temperature results in the detection of bands whose frequencies are close to the pure liquid compound, suggesting a weak molecular interaction through silanol groups whose IR band appears to be strongly perturbed. A closer analysis of the low frequency region of the spectra (not reported) also shows some shift towards lower frequencies of the C–Cl stretching bands, affected by adsorption. Pyridine co-adsorption under these conditions still leads to the detection of Lewis sites; therefore, further evidencing a main interaction of the organic molecule with OH groups. On the other side, based on these data, we cannot rule out also the coordination of a fraction of 1,2DCP molecules with Lewis sites, but pyridine as a strong base can actually displace these molecules, which can be still detected, weakly interacting with the surface (Figure 3).

Also, the analysis of the high frequency region of the spectrum evidenced two groups of acidic OH groups: One fraction more strongly perturbed (i.e., shifted towards lower wavenumbers) by the interaction with pyridine, another fraction that just shifted by few cm^−1^, which is likely due to hydroxyl interacting with 1,2DCP (evidenced in Figure 3b).

**Figure 4:**
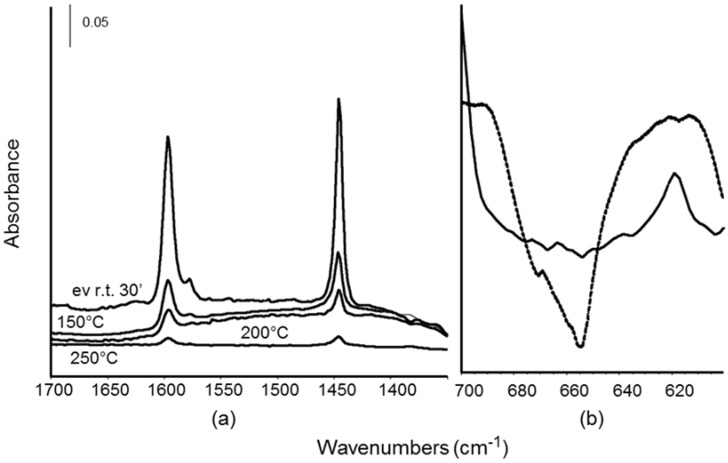
FT-IR subtraction spectra: (**a**) Surface species arising from pyridine adsorption over 30Co/SBA catalyst after pretreatment in 1,2DCP at 180 °C (the spectrum of the surface after 1,2DCP reaction has been subtracted); and, (**b**) enlargement of the low frequency spectral region of the 30Co/SBA sample after pretreatment in 1,2DCP at 180 °C (solid line) after pre-treatment at high temperatures (broken line).

Afterward, the catalyst has been treated at 180 °C in the presence of 1,2DCP vapor and pyridine adsorption has been carried out after a short outgassing. In this case, Lewis sites that are characterized by band above 1600 cm^−1^ disappear, possibly occupied by some transformation products that are still bound at the surface (Figure 4, subtraction spectra). Such intermediate species can have an alkoxy structure, as already suggested by investigating chlorinated compounds conversion [1,14]. Unfortunately, bands unambiguously characterizing alkoxides (i.e., CC/CO stretchings) are masked by the strong silica structure absorptions in the range 1000–1200 cm^−1^. Nevertheless, we have been able to detect some weak, newly formed bands in the subtraction spectra together with a multiplicity of bands in the CH deformation and stretching spectral region (spectra not reported). Under these conditions no reaction products are detected in the gas phase and structural bands due to Co_3_O_4_, whose lattice oxygen is involved in the oxidation process through the Mars van Krevelen mechanism [20,23], are almost unperturbed, as deduced from the spectrum reported in Figure 4b. In this subtraction spectrum (i.e., (spectrum after treatment with 12DCP at 180 °C and adsorbed py)—(spectrum of the oxidized surface)), the main band related to oxidized CoO_x_ centred near 660 cm^−1^ does not appear, pointing out that no perturbation of this features occurs below 200 °C. For comparison, we reported also a subtraction spectrum that were recorded after oxidation of 1,2DCP above 400 °C, which clearly shows a negative band corresponding to the erosion of lattice oxygen in the oxidation process.

Over the same 30Co/SBA catalyst we have performed the conversion of a mixture of 1,2DCP and air directly in the IR cell under static conditions, in the entire temperature range 100–500 °C.

**Figure 5:**
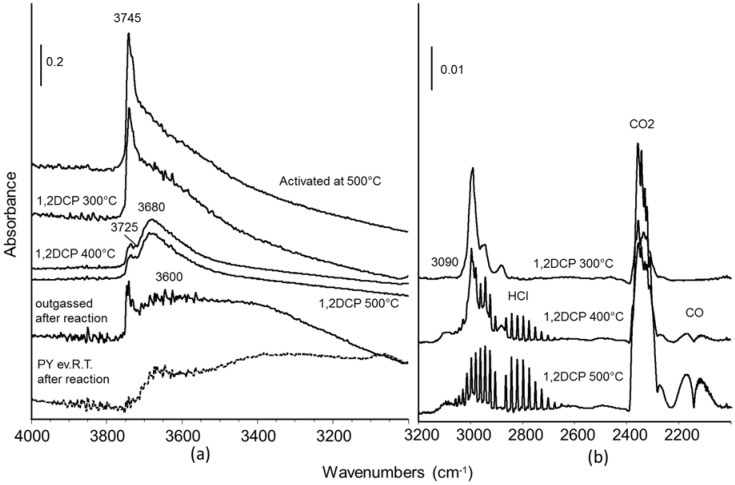
FT-IR spectra of: (**a**) 30Co/SBA hydroxy groups; and, (**b**) FT-IR spectra of gas phase species arising from 1,2DCP reaction.

The results have been discussed in detail elsewhere [20], and are only shortly summarized here. At 300 °C, isolated silanol groups at 3745 cm^−1^ are still detected in the spectra of surface species, although slightly reduced in intensity due to the interaction with the organic reactants and products (Figure 5a). In the gas phase spectra at the same temperature, the main bands are those owing to the chlorinated compound in the CH stretching region (2980–2800 cm^−1^), while few very weak bands already appear above 3000 cm^−1^ and around 2800 cm^−1^ (Figure 5b).

Above 300 °C, both gas phase and surface spectra significantly change. In the gas phase, new spectral features are detected: Sharp rotovibrational bands due to HCl gas in the range 2900–2600 cm^−1^, a complex absorption of gaseous CO_2_ at 2350 cm^−1^ (with a shoulder at ca 2300 cm^−1^ assigned to natural occurring isotopic CO_2_), and a rotovibrational band of gaseous CO at 2143 cm^−1^. Weak bands are still detected at 3090 cm^−1^ (ν = CH) and in the low frequency region, typical of different isomers of chloropropene arising from dehydrochlorination process. These findings are consistent with results that were reported in an early study on the catalytic 1,2DCP conversion on silica alumina, tested in a flow reactor in the range 130–530 °C [24].

CO still detected at 500 °C is likely formed as consequence of the incomplete combustion of 1,2DCP, which is present in very high amount in the IR cell under our experimental conditions and/or some homogeneous reaction. It is worth noticing that between 400 and 500 °C, for instance, when HCl production is evident in the gas phase, the intensity of the silanol band strongly decreases, another very weak shoulder is noticed at 3725 cm^−1^, and the main component of the spectrum is a broad and complex absorption centred at 3680 cm^−1^ ca, which can be assigned to H-bonded OH groups. Clearly, gaseous HCl, together with small amount of water vapour arising from combustion, partially affect surface OH groups. The following outgassing at room temperature removes all of the gas phase species, but only partially restores isolated silanols. The shoulder at 3725 cm^−1^ and a broad absorption signal extending from 3700 to 3300 cm^−1^ is still observed. Two components are likely centered just at about 3600 and 3400 cm^−1^ (Figure 5a).

Pure silica and silicalite interaction with HCl has been extensively studied by means of FT-IR and theoretical calculations by Pazé et al. [25,26]. These authors reported that at ca −63 °C HCl adsorption led to the gradual formation of hydrogen-bonded adducts SiOH–(HCl)_n_, eroding all of the exposed silanol groups at increasing HCl pressure and giving rise to a complex absorption below 3750 cm^−1^. In particular, the component at 3725 cm^−1^ has been assigned by theoretical calculation to the adduct Si–OH–(HCl). Moreover, a strong absorption band that was centered at 2788 cm^−1^ is associated with HCl molecules interacting with the siliceous framework. The reduction of the silanol band intensity and the following formation of a broad absorption at 2510 cm^−1^ have also been reported by Mosallanejad et al. for the H-bonding interaction of CuZSM-5 with HCl at room temperature [27].

Under our experimental conditions, some of these features are not evident, and this is likely due to the low amount of HCl that was formed during DCP combustion and to the co-presence of reaction products at high temperatures. However, we can explain our results by the interaction of HCl with a fraction of silanols through H-bonds, occurring at high temperature and leading to a limited shift of the corresponding OH stretching band.

**Figure 6:**
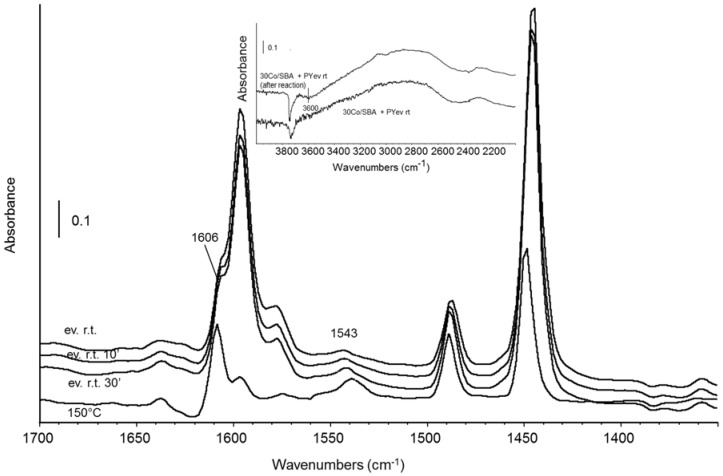
FT-IR subtraction spectra of surface species arising from pyridine adsorption and desorption over the 30Co/SBA catalyst after pretreatment in 1,2DCP/air in the range 50–500 °C. Inset: High frequency region.

Pyridine adsorption was performed over the surface after 1,2DCP reaction and a following outgassing step. The main bands are the same as discussed above for pyridine interaction with the fresh surface, but two significant differences are noticeable (Figure 6).

(i) A new weak band at 1543 cm^−1^ and a shoulder at 1635 cm^−1^ correspond to pyridinium ion formation. This evidence indicates Bronsted acidity at the surface, which is able to protonate pyridine after reaction and is induced by the contact with HCl at high temperature. In the high frequency region, we can detect a negative band due to silanol species at 3745 cm^−1^, together with another negative component that is broadly centered at 3600 cm^−1^, which may be due to another family of more acidic OHs interacting with pyridine through proton transfer (Figure 6). Pyridinium ions resist outgassing up to 150 °C.

(ii) The shoulder assigned to pyridine coordinated over Lewis sites at 1606 cm^−1^ is already detected at room temperature, its relative intensity being higher than in the spectrum of pyridine adsorbed over the fresh surface already at room temperature (Figure 2 vs. Figure 6). Therefore, after reaction, an increased amount of cobalt ions are exposed at the catalyst surface, acting as Lewis sites, which is maybe due to a limited etching of CoO_x_ particles. These findings are in agreement with the increase in Lewis acidity reported by Gracia et al. over regenerated Zr-SBA-15 catalysts, which is explained by the generation of new extraframework (O)ZrCl_x_ species [28].

1,2DCP conversion has been also tested in the IR cell without air, i.e., consuming the catalyst lattice oxygen to oxidize the organic substrate in the temperature range 100–500 °C. After this pretreatment, and following pyridine adsorption, the detection of pyridinium ions is confirmed, together with pyridine coordinated over Lewis sites and these surface species were resisting outgassing up to 250 °C. The band due to pyridine interacting with acidic silanol (i.e., below 1600 cm^−1^) is very weak, which is possibly due to the formation of surface heavy products and coke, hindering the availability of OH groups.

**Figure 7:**
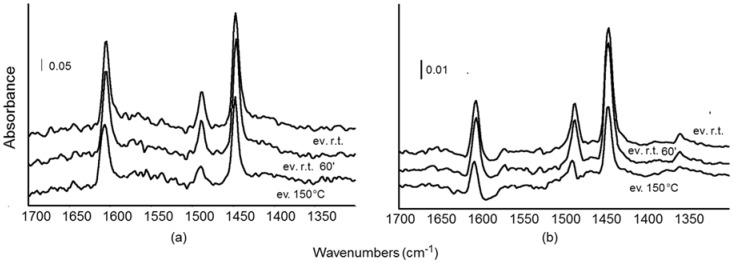
FT-IR subtraction spectra of surface species arising from pyridine adsorption and desorption: (**a**) Over the fresh reference CoO_x_ catalyst and (**b**) over reference CoO_x_ catalyst after pretreatment in 1,2DCP/air in the range 50–500 °C.

The same experimental procedure that was performed over the pure CoO_x_ sample does not reveal any Bronsted acidic sites (Figure 7), in spite of the formation of HCl and oxidation products in the gas phase during 1,2DCP oxidation in the IR cell. This effect should point out the direct implication of the SBA-support in the formation of new Bronsted acidic sites. A very small shift to higher frequencies can be noticed for the band of pyridine coordinated on Co ions in the spectrum collected after 1,2DCP treatment (from 1606 to 1608 cm^−1^), which is possibly related to an increase in the acidity strength of these sites.

**Figure 8:**
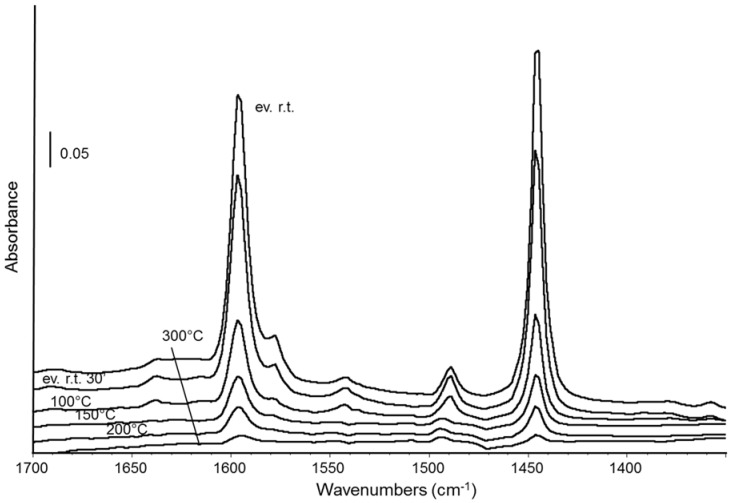
FT-IR subtraction spectra of surface species arising from pyridine adsorption over SBA-15 support after pre treatment in 1,2DCP in the range 50–500° C.

In order to further investigate this point, 1,2DCP conversion and the following pyridine adsorption have been carried out over the pure SBA-15 support. Heating 1,2DCP in the presence of SBA-15 leads to the formation of dehydrochlorination products (band at 3060 cm^−1^) and HCl (rotovibrational profile at 3000–2500 cm^−1^) in the low temperature range and up to 500 °C. As a consequence, the catalyst surface is in contact with a quite complex products mixture. After this pretreatment, pyridine adsorption evidences once again Bronsted acidity (Figure 8), but pyridinium ion disappears upon outgassing between 100 and 150 °C, i.e., at a temperature where it is well detectable over the 30Co/SBA catalyst. Moreover, the relative intensity of this band is slightly reduced in comparison to the 30Co/SBA catalyst.

Tsyganenko et al. also reported the formation of Bronsted centers over silica treated with (Lewis) acidic molecules, i.e., SO_2_ and NO_2_. They assigned this effect to the interaction of silanol groups with Lewis acid, increasing the acidity of the proton. These authors underlined that weak protonic acid, such as H_2_S, while inducing an increase in silanol acidity, are not able to provoke the protonation of dimethylpyridine [29,30]. In our very demanding reaction conditions, Bronsted acidity could be due to a fraction of molecularly adsorbed HCl, although the analysis of the high frequency region of the spectra does not clearly reveal such features, as discussed above [25,26,27]. On the other side, HCl can interact with the siliceous framework and we cannot rule out the breaking of some Si–O–Si bond in the mesoporous support, leading to the formation of acidic groups that are able to protonate pyridine. Therefore, the comparison between results that were obtained following pyridine adsorption on both 30Co/SBA catalyst and SBA support suggests a synergistic effect between cobalt oxide and silica to stabilize and enhance Bronsted acidity.

**Scheme 1:**
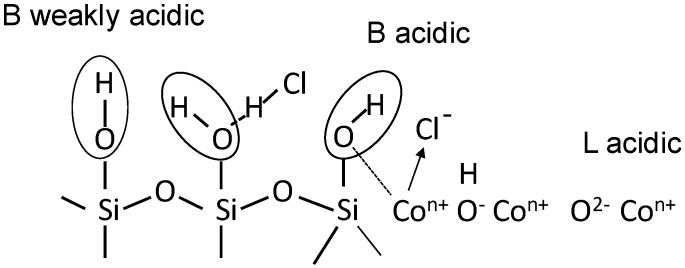
Functional groups at the catalyst surface (B = Bronsted sites, L = Lewis sites)

Correspondingly, SEM-EDX analysis point out that CoCl_x_ species can also be formed on the 30Co/SBA catalyst, thus suggesting that HCl may dissociatively adsorb at the catalyst surface with the participation of Co ions. Cobalt ions, whose acidity can be further increased by an electron whitdrawing effect of the chlorine atom, can assist the deprotonation of some surface OH groups (Scheme 1).

In sum, spectroscopic data indicate that in our conditions both Lewis and weakly acidic silanol are involved in the interaction with 1,2DCP, coordination with Lewis sites being relevant at temperatures above 200 °C. After the pretreatment in 1,2DCP up to 500 °C, in air or in vacuum, some Bronsted acidic sites that are able to protonate pyridine were formed at the surface of the used catalyst, together with weakly acidic silanols, as indicated by adsorption of pyridine. These data are confirmed by NH_3_-TPD results, which show that the total amount of acidic sites per gram is increased in the used catalyst after activity tests in the flow reactor.

The formation of new acidic groups can be related to the generation of HCl in the gas phase at high temperature and its following (dissociative) adsorption at the catalyst surface, assisted by Co ions but also to the increased acidity of a fraction of surface OHs due to an electron withdrawing effect of nearby Co-chloride species. At the same time, Lewis acidity from Co ions and weakly acidic silanols from the SBA support are still detectable.

This is a likely picture of the evolution of the working surface during Cl-VOC oxidation, i.e., in the presence of HCl, residual chlorinated organics, water, and CO_x_. Changes in surface acidic properties of the catalyst may favor Cl-VOC molecules adsorption and following activation at the catalyst surface.

Highly dispersed Co oxide on the mesoporous support and Co-chloride or oxychloride particles together with the presence of several families of acidic sites, having different strength and nature and originated from the conditioning effect of reaction products, may explain the high activity of this catalyst in chlorinated alkanes combustion.

## Supplementary Materials

The following are available online at http://www.mdpi.com/1996-1944/11/6/912/s1. Figure S1: N_2_ adsorption-desorption isotherms of the 30Co/SBA catalyst and the pure SBA-15 support and pore size distribution, Figure S2. XRD pattern of the 30Co/SBA catalyst. Table S1: Textural properties of the SBA-15 support and the 30Co/SBA catalyst.
