# Supplementary material for: Surface Characterization of Mesoporous CoOx/SBA-15 Catalyst upon 1,2-Dichloropropane Oxidation
Source: Materials (Basel). 2018 May 29;11(6):912. doi: 10.3390/ma11060912 (PMC6025381; doi:10.3390/ma11060912)
Supplement: Supplementary file 1 [file materials-11-00912-s001.pdf]

materials

Supplementary material

Surface characterization of mesoporous CoOx/SBA-15 catalyst upon 1,2-dichloropropane oxidation

Elisabetta Finocchio [1,*], Jonatan Gonzalez-Prior [2], Jose Ignacio Gutierrez-Ortiz [2], Ruben Lopez-Fonseca [2], Guido Busca [1] and Beatriz de Rivas [2,*]

1. Dipartimento di Ingegneria Civile, Chimica e Ambientale (DICCA), Università degli Studi di Genova, Via all'Opera Pia 15, I-16145 Genova, Italy; guido.busca@unige.it
2. Departamento de Ingeniería Química, Facultad de Ciencia y Tecnología, Universidad del País Vasco UPV/EHU, P.O. Box 644, E-48080 Bilbao, Spain; jonatan.gonzalez@ehu.es (J.G.-P.); joseignacio.gutierrez@ehu.es (J.I.G.-O.); ruben.lopez@ehu.eus (R.L.-F.)

* Correspondence: elisabetta.finocchio@unige.it (E.F.); beatriz.derivas@ehu.es (B.d.R.); Tel.: +39-010-353-2919 (E.F.); +34-94-601-5553 (B.d.R.)

Received: 4 May 2018; Accepted: 26 May 2018; Published: date

The SBA-15 support exhibited a type IV isotherm and a H1 hysteresis loop (Figure S2). The isotherm of the cobalt catalyst was relatively similar to that of the silica, thereby pointing out that the mesoporous structure was preserved after cobalt impregnation. However, the surface area and the pore volume decreased from 743 to 440 m^2 g^{-1} and from 0.8 to 0.59 cm^3 g^{-1} (Table S1). Interestingly, the bimodal pore distribution of the silica template, with pores of 40 and 62 Å, still remained after the deposition. This evidence was probably related to the fact that Co_3O_4 particles were preferentially deposited on the external surface of the support.

Table S1. Textural properties of the SBA-15 support and the 30Co/SBA catalyst.

Catalyst	BET, m^2g^{-1}	Pore Volume, cm^3 g^{-1}	Pore Diameter, Å	Co_3O_4 Crystal Size, nm
SBA-15	743	0.88	52	–
30Co/SBA	440	0.59	58	14

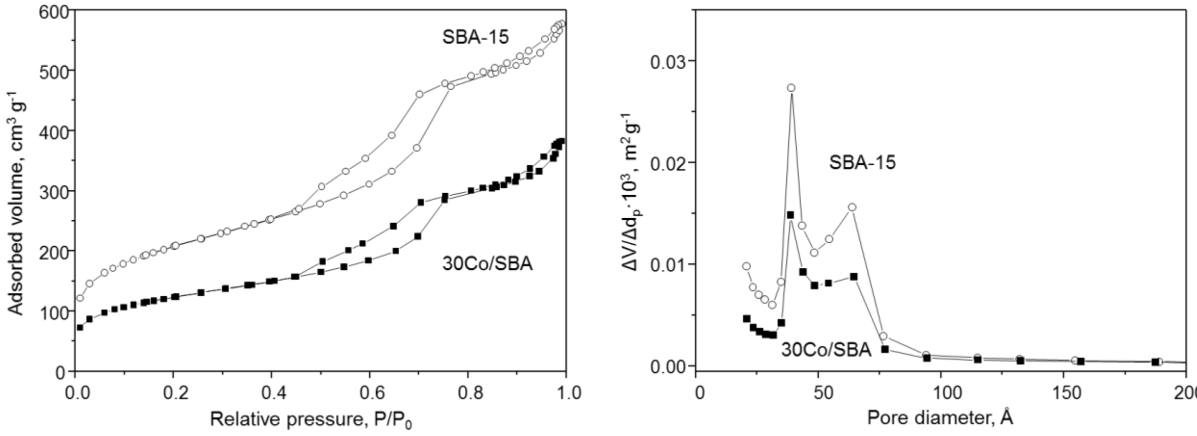

Figure S1. N_2 adsorption-desorption isotherms of the 30Co/SBA catalyst and the pure SBA-15 support (**left**) and pore size distribution (**right**).

The XRD (Figure S2) pattern of the 30Co/SBA15 catalyst showed reflection peaks located at 2θ 31.3º, 36.8º, 44.8º, 59.3º and 65.2º, which could be indexed to a pure cubic phase of Co_3O_4 spinel (JCPDS 78-1970). No diffraction peaks related to a CoO phase were detected. The average crystallite size,

estimated from the full width half maximum of the most intense diffraction peak by applying the Scherrer equation, was 14 nm.

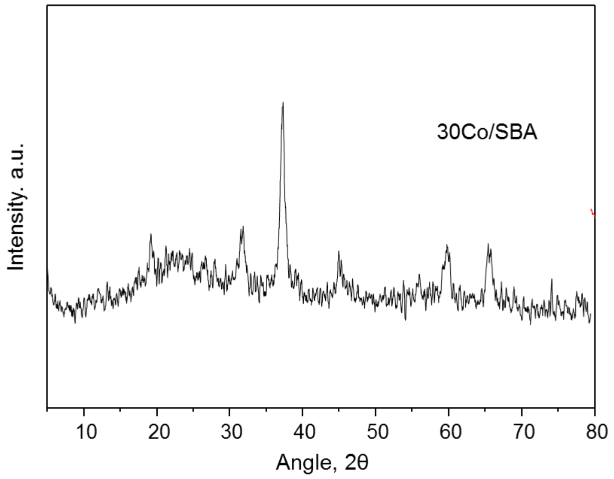

Figure S2. XRD pattern of the 30Co/SBA catalyst.

© 2018 by the authors. Submitted for possible open access publication under the terms and conditions of the Creative Commons Attribution (CC BY) license (http://creativecommons.org/licenses/by/4.0/).
